# Integration of network pharmacology and experimental validation to explore the pharmacological mechanism of andrographolide against asthma

**DOI:** 10.1186/s40643-025-00869-6

**Published:** 2025-04-08

**Authors:** Qian Yu, LiHong Zhu, XuChun Ding, YaFang Lou

**Affiliations:** Department of Pulmonary and Critical Care Medicine, Hangzhou TCM Hospital Affiliated to Zhejiang Chinese Medical University, Hangzhou, Zhejiang China

**Keywords:** Asthma, Andrographolide, Network pharmacology, Molecular docking, Th17 cell differentiation

## Abstract

Andrographolide (AG), one of the main active components of *Andrographis paniculata* (Burm.f.) Wall. ex Nees, has been proved to possess the pharmacological function of anti-inflammation in multiple disease including asthma. But the potential mechanism is still not clear. In this study, network pharmacology, molecular docking and experimental validation were utilized to explore the molecular mechanism of AG in the treatment of asthma. AG-related targets and asthma-related targets were screened by Swiss Target Prediction, DrugBank, STITCH, OMIM, Genecards and TTD databases. A protein–protein interaction (PPI) network was obtained through the STRING Database. The plug-in of “Network Analyzer” in Cytoscape 3.7.1 software was used to conduct the topological analysis. GO enrichment and KEGG pathway analysis were achieved by Metascape database and Bioinformatics platform. The target-pathway network was acquired by Cytoscape 3.7.1 software. The binding affinity between AG and the target genes was evaluated by Molecular docking with AutoDockTools 1.5.6. Flow cytometry was also used to verify the mechanism behind the treatment of asthma by AG, which was predicted in network pharmacology. In total, 38 targets were identified as potential targets of AG against asthma. The top 10 targets revealed by PPI are: IL-6, IL-1B, NFKB1, MMP9, CDK2, CREBBP, MAP2K1, JAK1, AR, PRKCA. GO and KEGG analysis showed that AG treatment of asthma mainly involved protein phosphorylation, peptidyl-serine phosphorylation, peptidyl-amino acid modification and other biological processes. The main signaling pathways are Th17 cell differentiation, JAK-STAT signaling pathway and PI3K-Akt signaling pathway. Molecular docking showed that AG has higher affinity with MMP9, PRKCA, JAK2, LTGAL and LRRK2. Flow cytometry showed that Th17 cell differentiation may be the potential target of AG in the treatment of asthma. This study successfully revealed the underlying target genes and mechanism involved in the treatment of asthma for AG, providing a reference and guidance for future mechanism research.

**Graphical abstract:**

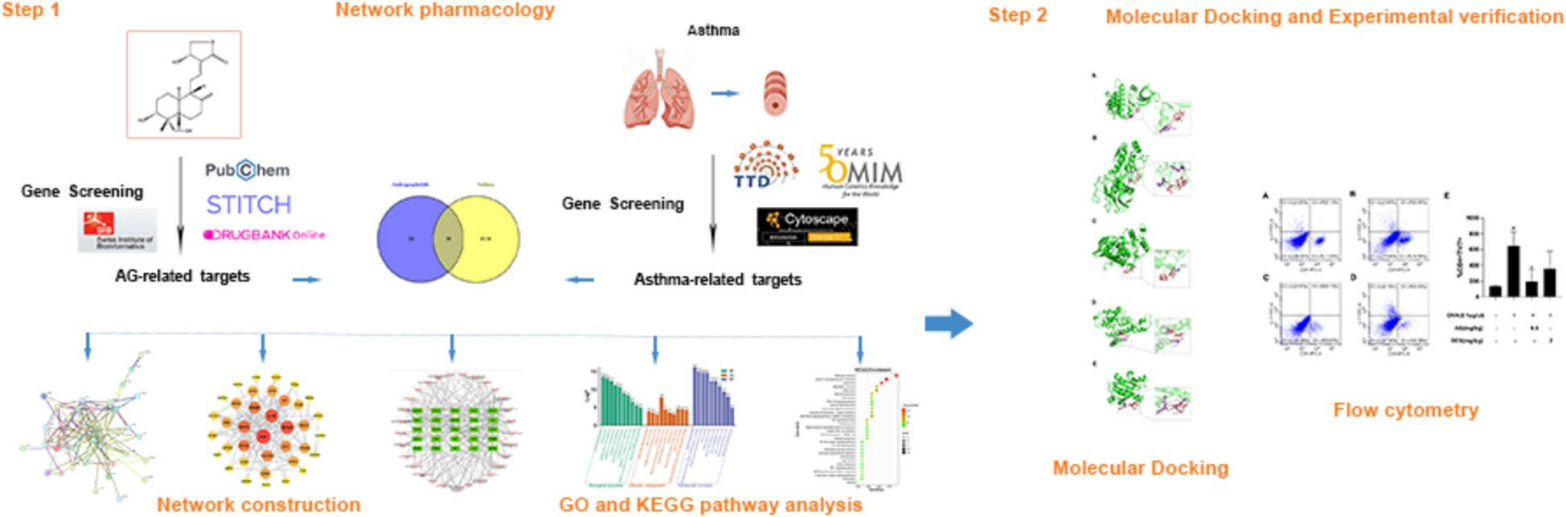

## Background

Asthma is a chronic inflammatory airway disease, characterized clinically by symptoms such as wheezing, shortness of breath, coughing, and chest tightness. The pathophysiological features of asthma include airway hyperresponsiveness (AHR), reversible airflow limitation, and airway remodeling. Currently, asthma affects more than 300 million people worldwide, with an increasing prevalence each year. Despite various therapeutic approaches, including inhaled corticosteroids, bronchodilators, and biologics, the mortality and morbidity associated with asthma remain alarmingly high, particularly in low- and middle-income countries, exacerbating health and social burdens (Global Initiative for Asthma 2023). Therefore, the exploration of new drugs or alternative management strategies for asthma is urgently needed.

Traditional Chinese Medicine (TCM) is a comprehensive discipline that integrates fundamental theories, Chinese Materia Medica, and clinical applications. Guided by the TCM concepts of holism and syndrome differentiation, each herb is utilized to restore and maintain the body’s homeostasis. Although often considered complementary, TCM has demonstrated significant therapeutic benefits in treating various diseases. *Andrographis paniculata* (Burm.f.) Wall. ex Nees, widely used in traditional Chinese medicine, and also in Ayurvedic medicine in India and traditional Thai medicine in Southeast Asia, is known for its effects in clearing heat and detoxicating, cooling the blood and reducing swelling and eliminating dampness. Beyond its anti-inflammatory and antioxidant properties, *A. paniculata* has shown remarkable therapeutic effects in treating infectious respiratory diseases (Selvaraj et al. 2022; Ratiani et al. 2022; Raj et al. 2023). Andrographolide (AG), one of the main active components of this herb, has been identified for its anti-asthmatic properties, which include immunoregulation, restoration of steroid sensitivity, and antioxidant effects (Liao et al. 2016; Peng et al. 2016; Yu et al. 2021; Sulaiman et al. 2024). However, the mechanisms underlying these effects are not yet fully understood and require further investigation.

It is confirmed that AG played an important role in controlling asthma: Prophylactic administration of AG prevented the progression of HDM-induced airway inflammation, remodeling and airway hyperreactivity by down-regulating Th2 cytokine gene expression and oxidative stress level (Sulaiman et al. 2024); AG also exerted protective function in the control and management of chemical-induced allergic asthma possibly by upregulating adherence junction proteins and activating p38/Nrf2 signaling (Sulaiman et al. 2018a, b); moreover, AG inhibited the expression of Th2 cytokine and serum level of OVA-specific IgE probably through inactivation of NF-kappaB pathway in asthmatic mice model (Zhang et al. 2009). These all indicate the anti-asthmatic function of AG and necessitate the further exploration of potential mechanism of AG in the treatment of asthma.

Network pharmacology is a powerful and integrative approach in drug design that encompasses multidisciplinary concepts such as systems biology, molecular biology, network analysis, and bioinformatics (Hopkins 2008; Muhammad et al. 2018). It has revolutionized the traditional “one disease-one target-one drug” paradigm, shifting towards targeting disease modules through the screening of small networks or disease modules (Nogales et al. 2022).In this study, we employ network pharmacology to explore the potential molecular mechanisms of AG in the treatment of asthma. With a series of bioinformatics analysis and experimental validation, including PPI network, GO and KEGG enrichment analysis, molecular docking and flow cytometry, we aim to identify the potential targets and pathways for AG in action of asthma. This study also shed a light for the further exploration of potential pharmacologic mechanisms of *A. paniculata* in treating asthma.

## Materials and methods

### Gene screening Andrographolide-related disease targets

“Andrographolide” was searched as the keyword in PUBCHEM database (https://pubchem.ncbi.nlm.nih.gov/) and the molecule structure and SMILES (Simplified molecular input line entry system) of the compound were obtained. Next, the SMILES number was imported into the Swiss Target Prediction database (http://www.swisstargetprediction.ch/). The potential targets were also searched from DrugBank (https://www.drugbank.ca/) and STITCH (http://stitch.embl.de/). Organism equal to Homo sapiens was limited. After removing the duplicated genes, andrographolide-related targets genes were returned.

### Gene screening of asthma-related targets

“Asthma” was searched as the keyword in the following databases, namely: OMIM (http://www.omim.org), Genecards (http://www.genecards.org) and Therapeutic Target Database (TTD) (http://db.idrblab.net/ttd) to obtain the disease related targets. After combining these targets and removing the repeated ones, we get the related targets of asthma.

### Retrieval of Venn diagram

Andrographolide and asthma related target genes were imported on the website (http://bioinfogp.cnb.csic.es/tools/venny/index.html). The overlapping genes of these two parts were obtained to draw the Venn Diagram.

### Construction of protein–protein interaction (PPI) network

The intersection of target genes obtained in the previous step were imported into the STRING Database (https://string-db.org) to build the PPI network. The species type was set to “Homo sapiens” and the minimum interaction threshold was set to “highest confidence” (> 0.4). The plug-in of “Network Analyzer”in Cytoscape 3.7.1 software was used to conduct the topological analysis to predict the key functional targets with large correlation.

### GO enrichment and KEGG pathway analysis

To study the biological function of potential targets in asthma, Metascape (https://metascape.org) database was used to collect GO analysis and KEGG data. GO enrichment analysis includes biological process (BP), cellular component (CC) and molecular function (MF). KEGG pathway analysis is used to select important and relative signaling pathways involved in biological processes. All the data were imported on the website of Bioinformatics platform (http://www.bioinformatics.com.cn/) for visual analysis.

### Construction of target-pathway network

Cytoscape 3.7.1 software was used to construct the target-pathway network to identify the interaction between the potential targets and signaling pathways enriched in KEGG pathway analysis.

### Molecular docking verification

The 3D structure of andrographolide was downloaded from PUBCHEM database and transformed to mol2 format by OpenBabel 2.4.0 software. The results were processed by AutoDockTools 1.5.6 software and stored in PDBQT format as the ligand file. The ternary crystal structures of target proteins were searched from PDB database (http://www.rcsb.org/) by screening and limiting to the species named “Homo sapiens”. The results were downloaded and stored in PDB format. PyMOL 1.7.2.1 was used for water removal and ligand separation. The results were hydrogenated and calculated by AutoDockTools 1.5.6 software and stored in PDBQT format as the protein receptor file. The ligand and receptor files were both imported into AutoDockTools 1.5.6 software again to run the molecular docking. Finally, the results were visualized and analyzed by PyMOL 1.7.2.1.

## Experimental validation

### Chemicals and reagents

Andrographolide (C_20_H_30_O_5_) was purchased from Herbpurify Co., Ltd. (Chengdu, China). The sample consisted of a white powder with a molecular weight of 350.45 and a purity of above 98% determined by HPLC analysis. Ovalbumin (OVA) was purchased from Solarbio Life Sciences (Beijing, China). The aluminum hydroxide gel was purchased from Thermo fisher scientific (Watham, MA, USA). Dexamethasone was purchased from Suicheng Pharmaceutical Co., LTD (Zhengzhou, China). Primary antibody (anti-mouse CD4, IL-17A), phorbol ester, Ionomycin, Brefeldin, permeabilization reagent were purchased from MULTISCIENCES (Hangzhou, China).

### Animals and care

Twenty male BALB/c mice, 6–8 weeks, weighing 20–25 g, were purchased from Laboratory Animal Centre of Zhejiang University of Traditional Chinese medicine. (SYXK [Zhe] 2021-0012). The mice were maintained in a specific pathogen-free (SPF) environment under controlled conditions of temperature (22 ± 1 °C), humidity (50%), 12 h light–dark cycles and free access to food and water. All efforts were made to ameliorate the welfare and minimize animal suffering.

### Animal groups and model

Animals were randomly divided into 4 groups: a control group, an OVA model group, AG (0.5 mg/kg) group and Dexamethasone (DEX) (2 mg/kg) group. The OVA models were established referring to the method described in our previous study (Yu et al. 2021). Animals were sensitized by intraperitoneal injection of 20 μg OVA in 200 μl and an equal volume of aluminum hydroxide on day 1, day 7 and day 14, respectively. Starting 21 days after the second sensitization, mice were challenged with atomized OVA (1% OVA dissolved into phosphate-buffered saline-PBS) for 30 min each day for 7 days. The control mice were treated with 0.9% physiological saline. The mice were separately intraperitoneally administrated AG at different concentrations and DEX 1 h before each challenge.

### Sample collection

At 24 h after the last drug administration, the mice were anesthetized by intraperitoneal injection of Zoletil (80 mg/kg). The lung tissue was collected.

### Flow cytometry

The cells collected from lung tissue homogenate were adjusted to the density of 1 × 10^6^/ml. 50 ng/ml of phorbol ester, 1 µg/ml of Ionomycin, 1 µg/ml of Brefeldin and RPMI 1640 culture medium with 10% newborn calf serum were put into the cells separately. After 6 h of cultivation in the incubator under the condition of 37 °C and 5% CO_2_, the cells were collected, fixed with 1% of Paraformaldehyde, washed by PBS and centrifuged for 5 min. After discarding the supernatant, 2 µl of anti-mouse CD4 antibody labeled with APC, permeabilization wash buffer and anti-mouse IL-17A antibody labeled with FITC were put into the cells. After incubation of 30 min and washing with PBS, the cells were resuspended and transferred to flow cytometer to detect the proportion of Th17 cells in the dark state.

## Results

### Target prediction and Venn diagram

**Fig. 1:**
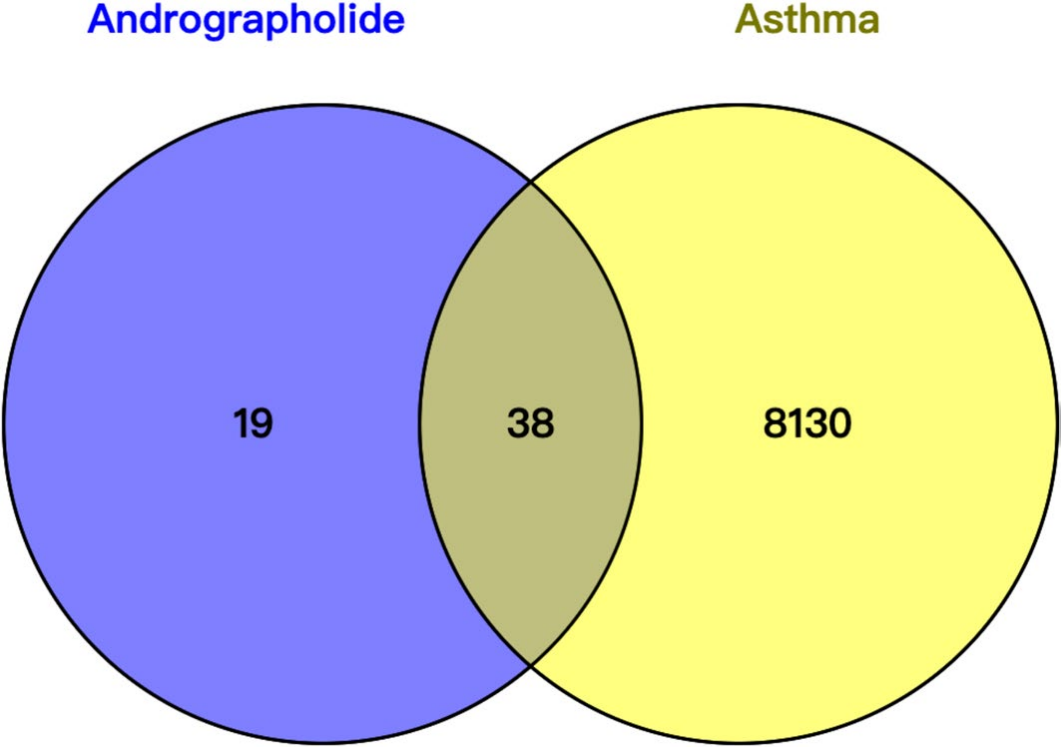
Venn diagram showing the targets of AG and asthma

A total of 57 andrographolide-related targets were collected from SwissTargetPrediction, DrugBank and STITCH databases, which were intersected with 8168 asthma-related targets collected from the OMIM, Genecards databases. 38 targets were identified as potential targets of andrographolide against asthma (Fig. 1, Table 1).

**Table 1:** Potential targets of andrographolide against asthma

Gene symbol	Uniprot ID	Protein name
PRKCA	P17252	Protein kinase C alpha
PRKCD	Q05655	Protein kinase C delta
IL6	P05231	Interleukin-6
ADAM17	P78536	ADAM17
GPBAR1	Q8TDU6	G-protein coupled bile acid receptor 1
FABP1	P07148	Fatty acid-binding protein
AR	P10275	Androgen receptor
PDCD4	Q53EL6	Programmed cell death protein 4
EPHX2	P34913	Epoxide hydratase
JAK1	P23458	Tyrosine-protein kinase JAK1
BRD4	O60885	Bromodomain-containing protein 4
CREBBP	Q92793	CREB-binding protein
PYGL	P06737	Liver glycogen phosphorylase
DPP4	P27487	Dipeptidyl peptidase IV
PRKCE	Q02156	Protein kinase C epsilon
PDE10A	Q9Y233	Phosphodiesterase 10A
PGR	P06401	Progesterone receptor
CDK2	P24941	Cyclin-dependent kinase 2
CDK1	P06493	Cyclin-dependent kinase 1
GABRB3	P28472	GABA-A receptor; beta-3
GABRA2	P47869	GABA-A receptor; alpha-2
ROCK1	Q13464	Rho-associated protein kinase 1
OPRM1	P35372	Mu opioid receptor
NR3C2	P08235	Mineralocorticoid receptor
CHEK1	O14757	Serine/threonine-protein kinase Chk1
ERN1	O75460	Serine/threonine-protein kinase/endoribonuclease IRE1
G6PD	P11413	Glucose-6-phosphate 1-dehydrogenase
JAK2	O60674	Tyrosine-protein kinase JAK2
MAP2K1	Q02750	Dual specificity mitogen-activated protein kinase 1
MMP9	P14780	Matrix metalloproteinase 9
ACVRL1	P37023	Serine/threonine-protein kinase receptor R3
ITGAL	P20701	Integrin alpha L
CDK9	P50750	Cyclin-dependent kinase 9
ITGB2	P05107	Integrin alpha-L/beta-2
LRRK2	Q5S007	Leucine-rich repeat serine
IL1B	P01584	Interleukin-1B
NFKB1	P19838	Nuclear factor kappa B1
NFKB2	Q00653	Nuclear factor kappa B2

### PPI network construction

**Fig. 2:**
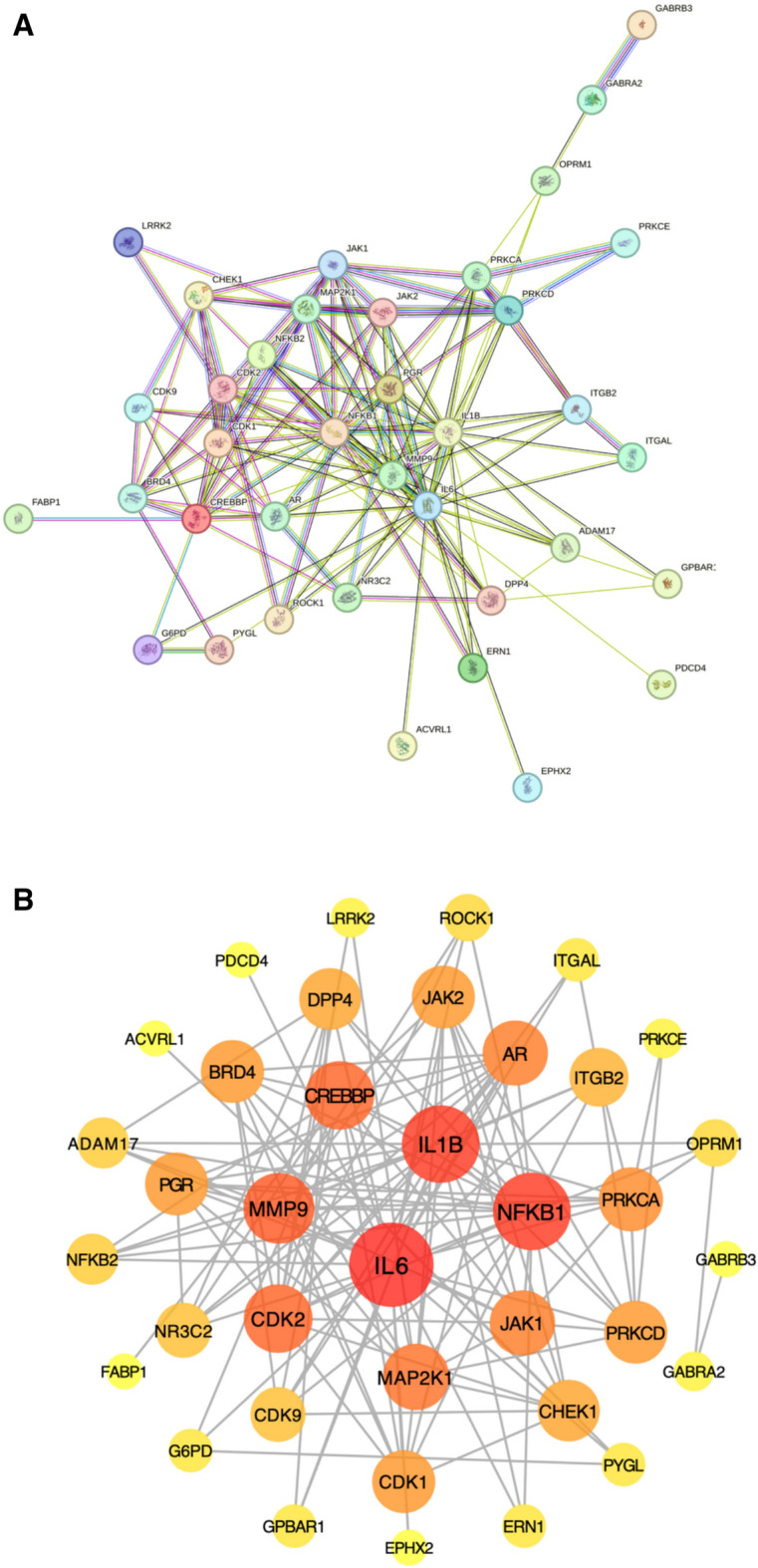
PPI network of potential target genes. **A** The nodes represent proteins and the edges represent the interaction between the proteins. **B** Core PPI network according to the degree value of each target. Darker node color indicates higher degree value

The information of the 38 targets were imported to the STRING database to construct the protein–protein interaction network. The PPI network was consisted of 38 nodes and 142 edges after hiding disconnected nodes in the network (Fig. 2A). The nodes represent proteins and the edges represent the interaction between the proteins. To obtain the core PPI network, the initial PPI network was imported in cytoscape 3.7.1. The plug-in of “Network Analyzer” was used to analyze and evaluate the PPI network according to the degree value of each target (Fig. 2B). Darker node color indicates higher degree value.

### GO and KEGG pathway enrichment analysis

**Fig. 3:**
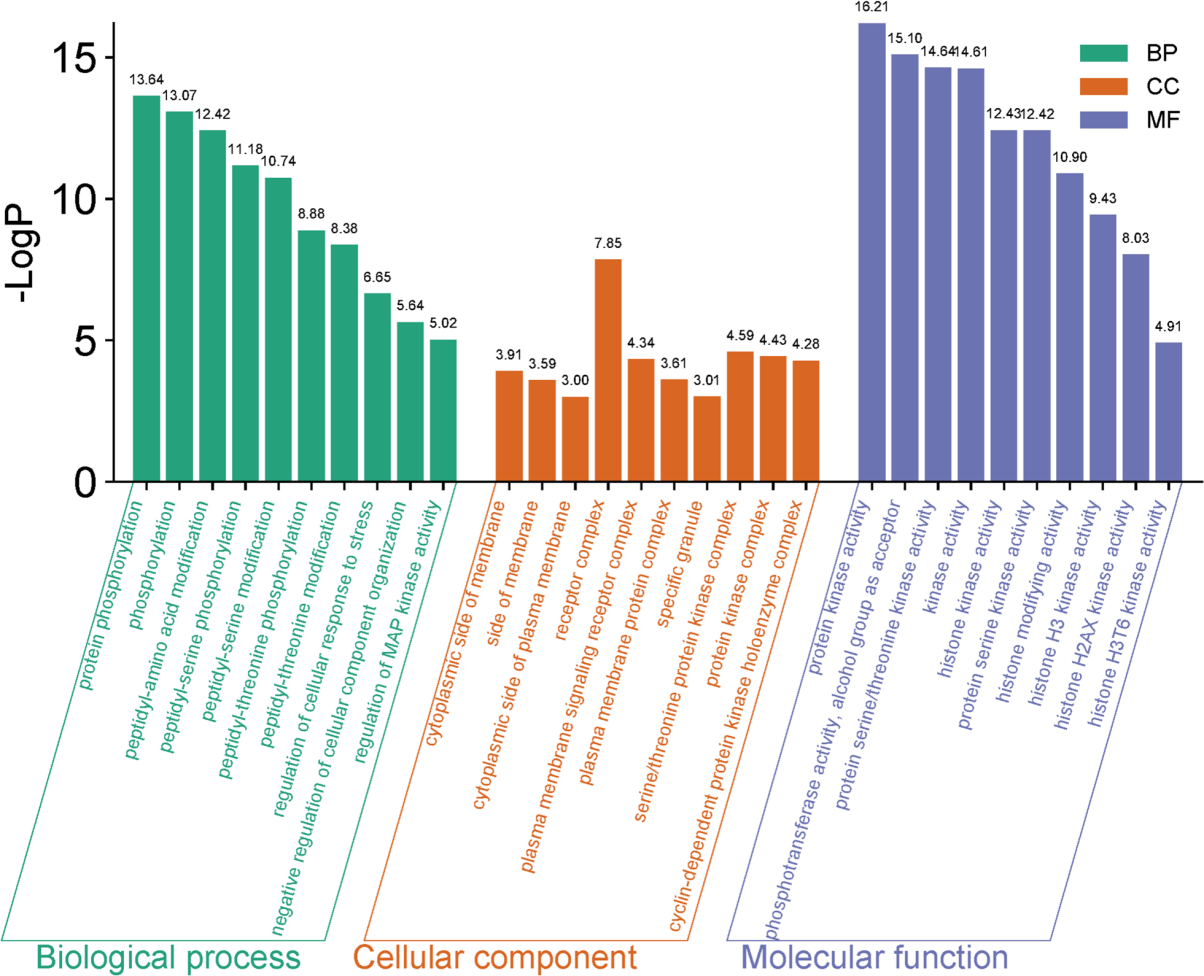
GO enrichment analysis. Top 10 BP, CC and MF are represented in green, orange and purple

We performed GO enrichment analysis to investigate the potential function of the 38 common targets and top 10 items of BP, CC, and MF were taken to make a visual bubble diagram (Fig. 3). The top 5 enriched BP mainly involves in protein phosphorylation, phosphorylation, peptidyl-amino acid modification, peptidyl-serine phosphorylation and peptidyl-serine modification. The top 5 enriched CC were cytoplasmic side of membrane, side of membrane, cytoplasmic side of plasma membrane, receptor complex and plasma membrane signaling receptor complex. The top 5 enriched MF were protein kinase activity, phosphotransferase activity, alcohol group as acceptor, protein serine/threonine kinase activity, kinase activity and histone kinase activity.

**Fig. 4:**
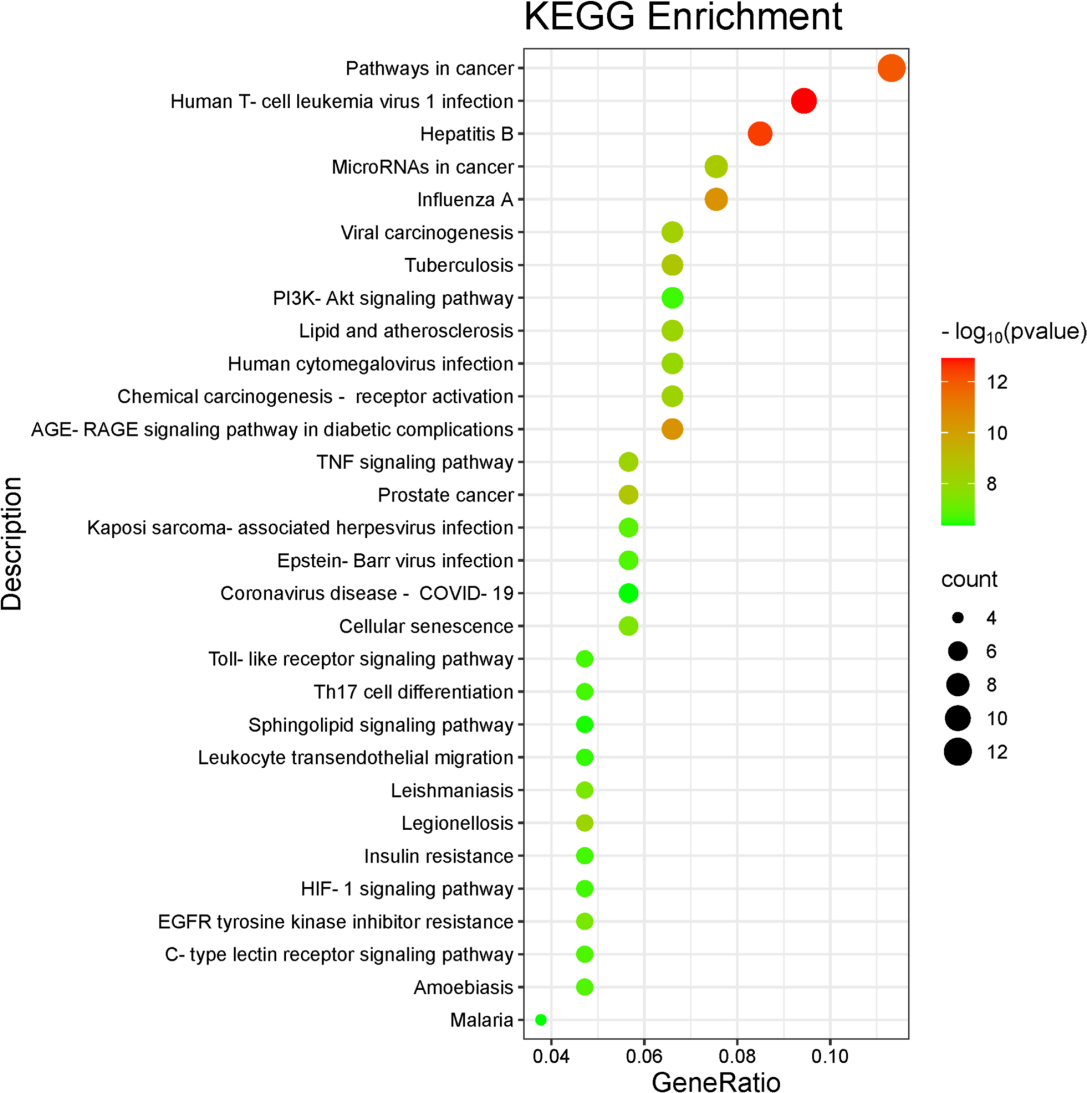
Bubble chart of the top 30 pathways based on KEGG enrichment analysis

In order to explore the possible mechanism of andrographolide in the treatment of asthma, we performed KEGG enrichment analysis. 30 signaling pathways were screened and shown in the bubble diagram (Fig. 4), which mainly involve Th17 cell differentiation, PI3K-Akt signaling pathway, JAK-STAT signaling pathway, TNF signaling pathway, HIF-1 signaling pathway, Toll-like receptor signaling pathway and so on.

### Target-pathway network analysis

**Fig. 5:**
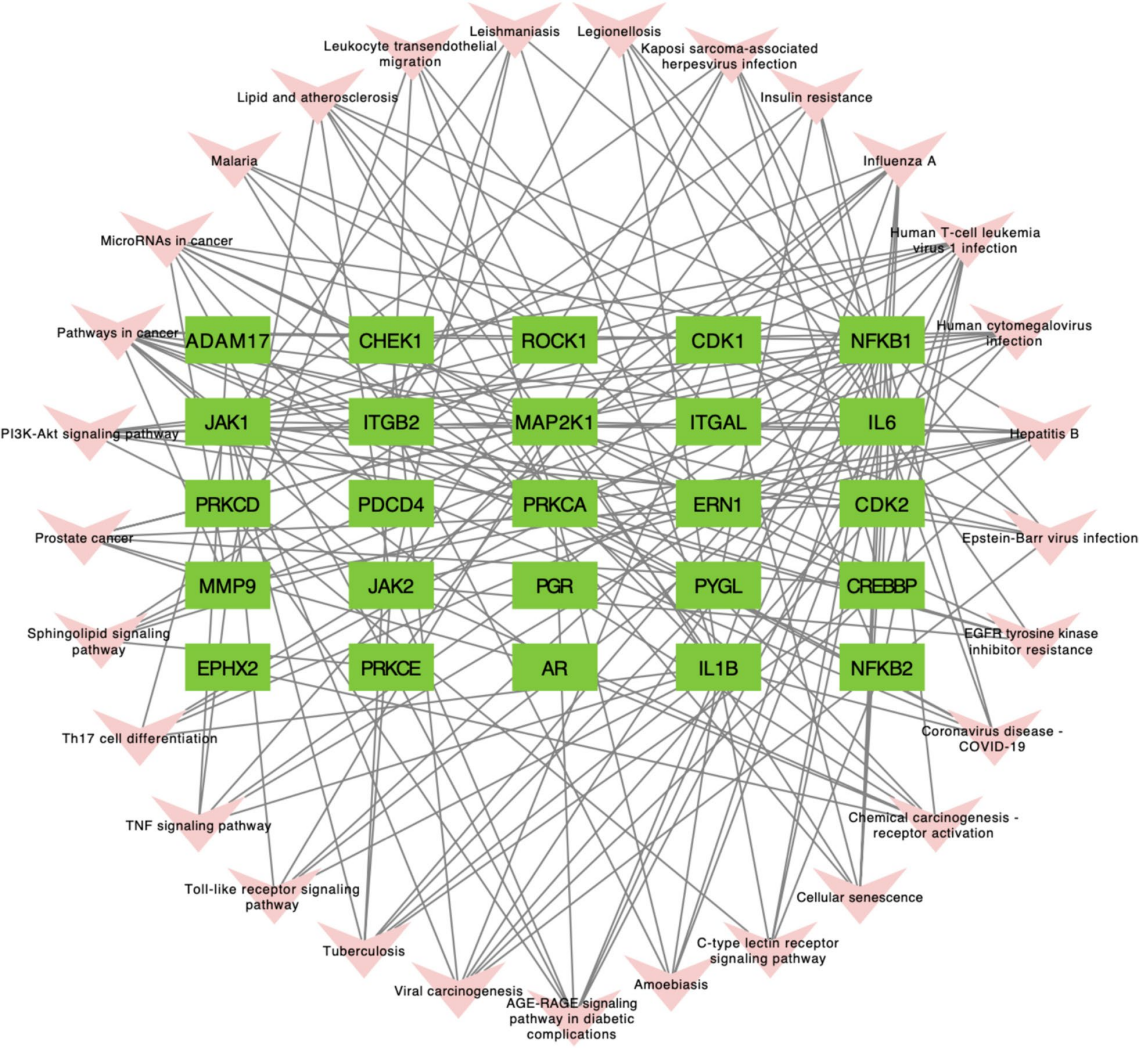
Target-pathway network analysis. Pink arrows represent the pathways. Green rectangles represent the targets

The target-pathway network analysis was constructed with cytoscape 3.7.1 software. The results showed that 25 core targets and 29 KEGG signaling pathways were highly relevant in the treatment of asthma by Andrographolide (Fig. 5). The results also showed that, among the top 29 pathways, by which AG inhibited asthma, Th17 cell differentiation, the JAK-STAT signaling pathway, and the PI3K-Akt signaling pathway should be the focus of further research. We also ranked the core targets according to the number of signaling pathways involved and found that NFKB1, IL6, MAP2K1, JAK1 and IL1B were top 5 target genes involved in a wide range of signaling pathways.

### Molecular docking

**Fig. 6:**
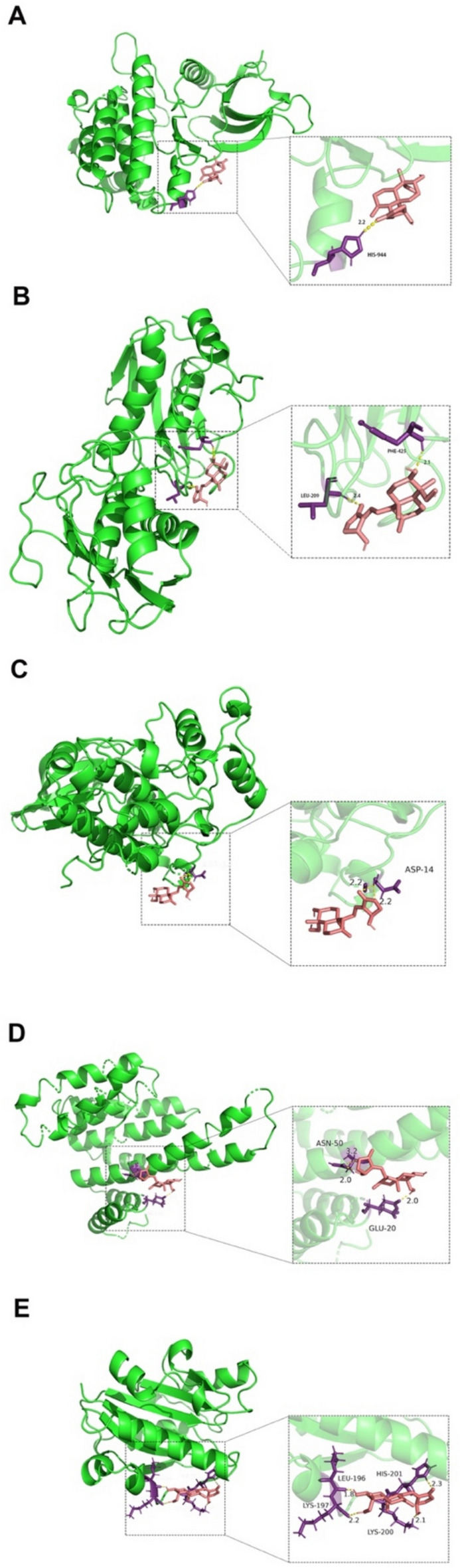
Molecular docking of target genes and AG. **A** JAK2-AG, **B** MMP9-AG, **C** PRKCA-AG, **D** LRRK2-AG, **E** ITGAL-AG

To validate the findings from network pharmacology, we utilized molecular docking to testify the binding affinity between AG and screened targets based on the results from PPI and KEGG. The information and details of receptor and ligand affinities are shown in Table 2. Figure 6 portrayed the docking images of three-dimensional structures of receptor-ligand affinities. The results showed that AG forms one hydrogen bond with His944-2.2A in JAK2 (Fig. 6A). Figure 6B shows that AG forms one hydrogen bond with Phe425-2.1A and Leu209-2.4A in MMP9. Figure 6C shows that AG forms two hydrogen bonds with Asp14-2.2A in PRKCA. Figure 6D shows that AG forms one hydrogen bond with Glu20-2.0A and two hydrogen bonds with Asn50-2.0A-3.2A in LRRK2. AG also could interact with Leu196-1.8A, Lys197-2.2A, Lys200-2.1A and His201-2.3A in ITGAL through one hydrogen bond (Fig. 6E).

**Table 2:** Binding energy (kcal/mol) of target genes and AG

No	Target	PDB ID	Binding energy
1	JAK2	7Q7K	− 5.09
2	MMP9	1GKD	− 5.17
3	PRKCA	6HOU	− 5.19
4	LRRK2	5MY9	− 5.24
5	ITGAL	1MJN	− 5.58

### AG targets Th17 cell differentiation

**Fig. 7:**
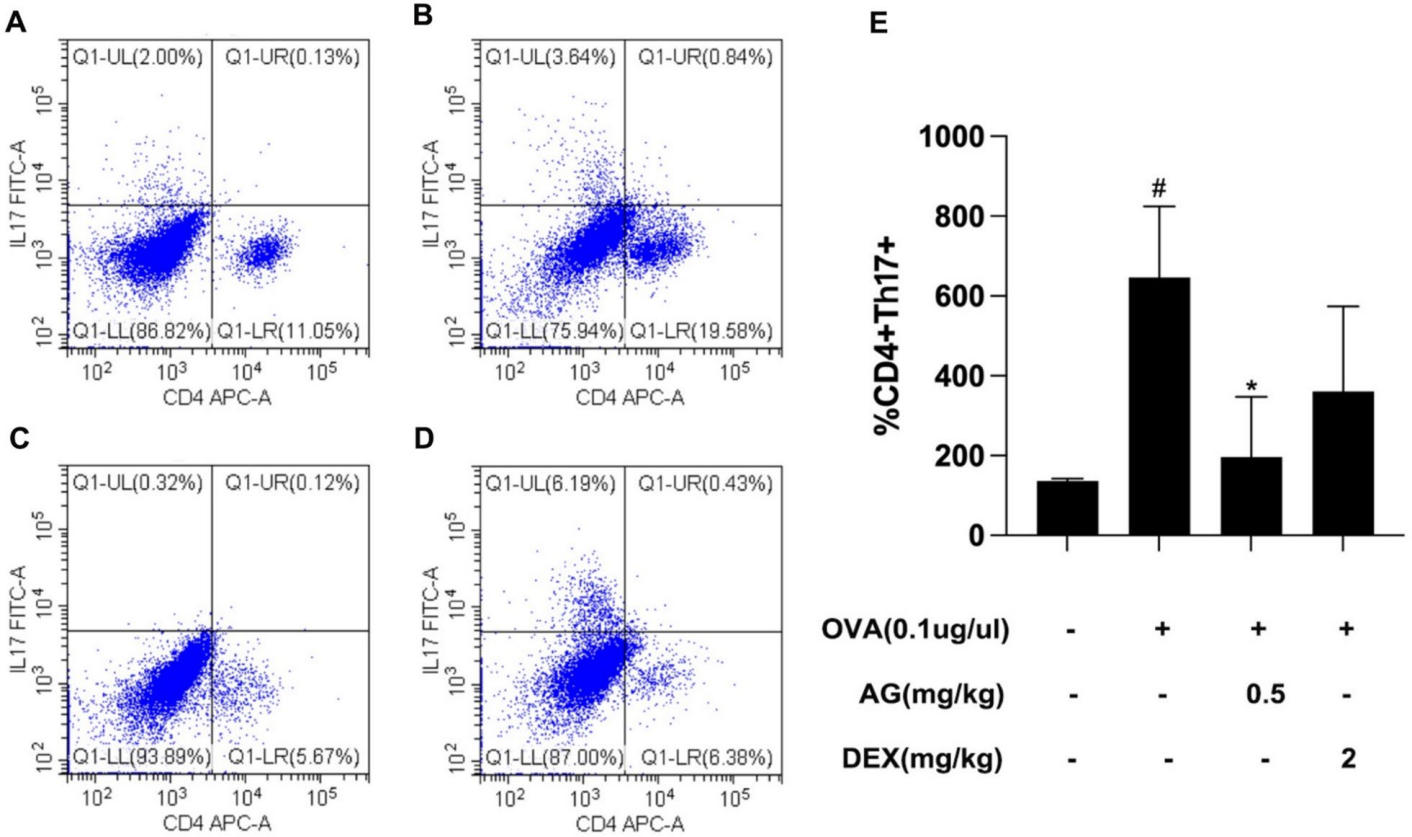
In vivo experimental verification of flow cytometry. Animals were randomly divided into 4 groups: control group (**A**), OVA group (**B**), AG group (**C**), DEX group (**D**), *n* = 5. OVA (0.1 μg/μl) treatment promoted the differentiation of Th17 cells, which was inhibited by AG. All values are expressed as the mean ± the standard deviation of triplicate tests. #*p* < 0.05 relative to the control group; *p < 0.05 relative to the OVA group

In order to verify the function of AG towards Th17 cell differentiation, we conducted the flow cytometry. The results showed that in Fig. 7 the percentage of differentiated Th17 cell was obviously increased in OVA-induced mice. Noticeably, AG decreased the differentiation of Th17 cell.

## Discussion

Asthma is a heterogeneous disease with multiple endotypes, each characterized by distinct pathophysiological mechanisms. For some asthmatic patients, anti-inflammatory therapy with corticosteroids proves ineffective. This challenge not only underscores the urgent need for new therapeutic options but also suggests the involvement of additional immunological processes and underlying mechanisms in asthma. *A. paniculata*, mainly used in traditional Chinese medicine, Ayurvedic medicine and traditional Thai medicine, is widely used in treating dyspnea and cough associated with lung-heat due to its properties of clearing heat and detoxication. Andrographolide (AG), one of the active components of this herb, has demonstrated significant anti-inflammatory effects, particularly in the treatment of asthma (Liao et al. 2016; Sulaiman et al. 2024). However, the mechanisms by which AG exerts its effects in asthma remain insufficiently explored.

TCM network pharmacology, first proposed and developed by Li, introduced a novel approach by bridging network science and TCM to discover the effective ingredients, new targets and potential mechanisms of traditional Chinese herb in disease treatment (Li 1999; Li et al. 2013). In the present study, we utilized network pharmacology, molecular docking and in vivo experiments to identify the potential targets and underlying mechanisms of AG in the treatment of asthma.

We collected AG-related targets from the SwissTargetPrediction, DrugBank and STITCH databases and asthma-related targets from the OMIM, Genecards and TTD databases. 38 common targets were identified as potential targets of AG against asthma. Next, a PPI network revealed interactions among these targets, highlighting the top 10 core targets: IL-6, IL-1B, NFKB1, MMP9, CDK2, CREBBP, MAP2K1, JAK1, AR, PRKCA. Numerous studies have established the close connection between asthma and these targets. For example, interleukin-6 (IL-6), a biomarker of systemic inflammation, plays a critical role in the pathological process of asthma: Patients with elevated plasma IL-6 concentration tend to exhibit worse lung function and more frequent asthma exacerbations compared to those with lower IL-6 concentration (Peters et al. 2016); Administration of IL-6 neutralizing antibody has been shown to improve AHR, reduce smooth muscle thickening, alleviate granulocytic inflammation and epithelial denudation, and inhibit innate immune responses (Chen et al.^2022^). Additionally, increased interleukin-1β (IL-1β) release contributes to increased inflammation and abnormal collagen remodeling in asthma (Osei et al. 2020). Study has also confirmed that the miR-9-5p/KLF5/IL-1β Axis could regulate airway smooth muscle cell proliferation and apoptosis in asthmatic mice (Xu et al. 2024). What’s more, as the key upstream protein of NF-κB signaling pathway, Nuclear Factor kappa B1 (NFKB1) involves in the expression of inflammatory cytokine and eosinophilia in the asthma exacerbation mouse model (Menzel et al. 2022). Besides, Matrix Metalloproteinase-9 (MMP-9), found in blood and bronchial alveolar fluid (BALF) of asthmatic patients, acted as a potential protease, promoting the release of IL-1β, another key inflammatory factor in asthma (Esnault et al. 2019). And also, Protein kinase A (PRKCA) has been shown to significantly reduce AHR, lung inflammation, mucus production, and infiltration of inflammatory mediators (Reber et al. 2012). Moreover, asthma improvement was observed through PKA-dependent inactivation of NF-κB pathway (Zhu et al. 2015). These findings suggest that these core target proteins may provide new insights into potential mechanisms of AG in treatment of asthma.

To further explore the underlying mechanisms of AG in asthma, we conducted GO and KEGG enrichment analysis. GO analysis indicated that the main BP involved was protein phosphorylation, the primary CC was receptor complex and the principal MF was protein kinase activity. Study has demonstrated the antagonistic role of fusion receptor complexes, such as the thymic stromal lymphopoietin receptor (TSLPR) and IL-17Rα, in the human TSLP signaling pathway, which mediates asthma via type 2 helper T cell responses (Verstraet et al. 2017). KEGG analysis revealed that the targets identified in this study were predominantly enriched in inflammation modulatory pathways, including PI3K-Akt and JAK-STAT signaling pathways, as well as Th17 cell differentiation. Numerous studies have documented the correlation between these pathways and asthma. For instance, the inhalation of PI3K inhibitor has been shown to reduce inflammatory responses and improve lung function in asthma (Campa et al. 2018). Multiple TCM formulas have also demonstrated inhibitory effects on asthma by inactivating the PI3K-Akt signaling pathway, including reductions in mucus production and cell infiltration, decreases in oxidative stress and airway inflammation, regulation of immunocytes balance and serum metabolism, improvement of AHR and partial reversal of airway remodeling (Jie et al. 2023; Nguyen et al. 2023; Yan et al. 2024). Furthermore, The JAK1/2 inhibitor Baricitinib has been shown to suppress eosinophil effector function by inhibiting eosinophil chemotaxis and respiratory burst, thereby limiting eosinophilia in allergen-induced airway inflammation (Luschnig et al.^2021^). Our previous study also revealed that AG could inhibit the expression of Th17 cell-related cytokines and the activation of JAK1/STAT3 signaling in asthmatic mice (Yu et al. 2021). All the results shed a light for the further exploration of the inhibitory function of AG in asthma.

The results of target-pathway network analysis demonstrated the involvement between target genes and a wide range of signaling pathways. Furtherly, molecular docking testified the binding affinity between AG and these target genes. Binding affinities for docking results of JAK2, MMP9, PRKCA, ITGAL and LRRK2 were all less than − 5 kcal/mol, indicating the good docking ability of these target genes with AG and their significant roles as potential binding targets.

Th17 cell is a kind of CD4 + T cell, expressing multiple cytokines such as IL-17A, IL-17F and IL-22 to regulate the progress of asthma. Th17 cell differentiation could induce the infiltration of airway neutrophils, increase mucus secretion and hyperplasia of airway smooth muscle, aggravate the collagen deposition of peripheral trachea and exacerbate the airway remodeling (Ramakrishnan et al. 2019). Inhibition of IL-17A, IL-17F and IL-17 receptor could ameliorate AHR and airway inflammation (Chenuet et al. 2017). Besides, IL-17A secretion also plays a key role in regulating neutrophilic airway inflammation and eventually leading to neutrophilic inflammation (Yang et al. 2023). The results of KEGG enrichment analysis have shown that Th17 cell differentiation could be the potential mechanism in AG regulating asthma. Moreover, the results of flow cytometry also demonstrated the inhibitory function of AG to Th17 cell differentiation in asthmatic mice. All these results showed that AG may target Th17 cell differentiation in the regulation of asthma.

## Conclusion

To summarize, this is the first study exploring the potential target genes and mechanisms of AG in the treatment of asthma, basing on network pharmacology, molecular docking and experimental verification. Our research shows that JAK2, MMP9, PRKCA, LRRK2 and ITGAL may be the potential targets of AG in treating asthma. In addition, AG may play a therapeutic role through multiple signaling pathways, especially Th17 cell differentiation. Overall, this study is expected to provide a reference for future mechanism research and guide the further development of AG in the treatment of asthma.

## Data Availability

The datasets utilized and examined in the present study can be obtained upon a reasonable request from the corresponding author.

## Abbreviations

AHR: Airway hyperresponsiveness
AG: Andrographolide
AR: Androgen receptor
BALF: Bronchial alveolar fluid
BP: Biological process
CC: Cellular component
CDK2: Cyclin-dependent kinase 2
CREBBP: CAMP response element binding protein binding protein
DEX: Dexamethasone
GO: Gene ontology
IL-17: Interleukin-17
IL-1B: Interleukin-1B
IL-6: Interleukin-6
ITGAL: Integrin alpha L
JAK-STAT: Janus kinase-signal transducer and activator of transcription
KEGG: Kyoto encyclopedia of genes and genomes
LRRK2: Leucine-rich repeat serine
MAP2K1: Dual specificity mitogen-activated protein kinase 1
MF: Molecular function
MMP-9: Matrix metalloproteinase-9
NFKB1: Nuclear factor kappa B1
NOD: Nucleotide-binding oligomerization domain
OVA: Ovalbumin
PGR: Progesterone receptor
PI3K-Akt: Phosphatidylinositol 3 kinase-protein kinase B
PKA: Protein kinase A
PPI: Protein–protein interaction
PRKCA: Protein kinase A
TCM: Traditional Chinese medicine
Th17: T helper 17
TSLPR: Thymic stromal lymphopoietin receptor
TTD: Therapeutic target database

## References

[CR2] Campa CC, Silva RL, Margaria JP, Pirali T, Mattos MS, Kraemer LR, Reis DC, Grosa G, Copperi F, Dalmarco EM, Lima-Júnior RCP, Aprile S, Sala V, Bello FD, Prado DS, Alves-Filho JC, Medana C, Cassali GD, Tron GC, Teixeira MM, Ciraolo E, Russo RC, Hirsch E (2018) Inhalation of the prodrug PI3K inhibitor CL27c improves lung function in asthma and fibrosis. Nat Commun 1:5232. 10.1038/s41467-018-07698-6 10.1038/s41467-018-07698-6 PMC6290777 30542075

[CR3] Chen S, Chen Z, Deng Y, Zha S, Yu L, Li D, Liang Z, Yang K, Liu S, Chen R (2022) Prevention of IL-6 signaling ameliorates toluene diisocyanate-induced steroid-resistant asthma. Allergol Int 1:73–82. 10.1016/j.alit.2021.07.004 10.1016/j.alit.2021.07.004 34332882

[CR4] Chenuet P, Fauconnier L, Madouri F, Marchiol T, Rouxel N, Ĺie Ledru A, Mauny P, Lory R, Uttenhove C, van Snick J, Iwakura Y, di Padova F, Ŕie Quesniaux V, é Togbe D, Ryffel B (2017) Neutralization of either IL-17A or IL-17F is sufficient to inhibit house dust mite induced allergic asthma in mice. Clin Sci (Lond) 20:2533–2548. 10.1042/CS20171034 10.1042/CS20171034 29026003

[CR5] Esnault S, Kelly EA, Johnson SH, DeLain LP, Haedt MJ, Noll AL, Sandbo N, Jarjour NN (2019) Matrix metalloproteinase-9-dependent release of IL-1 β by human eosinophils. Mediators Inflamm. 10.1155/2019/7479107 30906226 10.1155/2019/7479107 PMC6398033

[CR6] Global Initiative for Asthma (2023) Global strategy for asthma management and prevention updated July 2023. www.ginasthma.org

[CR7] Hopkins AL (2008) Network pharmacology: the next paradigm in drug discovery. Network pharmacology: the next paradigm in drug discovery. Nat Chem Biol 11:682–690. 10.1038/nchembio.118 10.1038/nchembio.118 18936753

[CR8] Jie XL, Luo ZR, Yu J, Tong ZR, Li QQ, Wu JH, Tao Y, Feng PS, Lan JP, Wang P (2023) Pi-Pa-Run-Fei-Tang alleviates lung injury by modulating IL-6/JAK2/STAT3/IL-17 and PI3K/AKT/NF-kappaB signaling pathway and balancing Th17 and Treg in murine model of OVA-induced asthma. J Ethnopharmacol 317:116719. 10.1016/j.jep.2023.116719 37268260 10.1016/j.jep.2023.116719

[CR9] Li S, Zhang B (2013) Traditional Chinese medicine network pharmacology: theory, methodology and application. Chin J Nat Med 2:110–120. 10.1016/S1875-5364(13)60037-0 10.1016/S1875-5364(13)60037-0 23787177

[CR10] Li S (1999) Possible relationship between traditional Chinese medicine ZHENG and molecular networks. The First Academic Annual Meeting of the China Association for Science and Technology

[CR11] Liao W, Tan WSD, Wong WSF (2016) Andrographolide restores steroid. Sensitivity to block lipopolysaccharide/IFN-γ-Induced IL-27 and airway hyperresponsiveness in mice. J Immunol 11:4706–4712. 10.4049/jimmunol.1502114 10.4049/jimmunol.1502114 27183596

[CR12] Luschnig P, Kienzl M, Roula D, Pilic J, Atallah R, Heinemann A, Sturm EM (2021) The JAK1/2 inhibitor baricitinib suppresses eosinophil effector function and restricts allergen-induced airway eosinophilia. Biochem Pharmacol 192:114690. 10.1016/j.bcp.2021.114690 34274356 10.1016/j.bcp.2021.114690

[CR13] Menzel M, Akbarshahi H, Mahmutovic I, Andersson C, Puthia M, Uller L (2022) NFkappaB1 dichotomously regulates pro-inflammatory and antiviral responses in asthma. J Innate Immun 14:182–191. 10.1159/000517847 34350857 10.1159/000517847 PMC9149445

[CR14] Muhammad J, Khan A, Ali A, Fang L, Yanjing W, Xu Q, Wei D (2018) Network pharmacology: exploring the resources and methodologies. Curr Top Med Chem 18:949–964. 10.1016/j.phymed.2024.155494 29600765 10.2174/1568026618666180330141351

[CR15] Nguyen V, Zhang Q, Pan F, Jin Q, Sun M, Tangthianchaichana J, Du S, Lu Y (2023) Zi-Su-Zi decoction improves airway hyperresponsiveness in cough-variant asthma rat model through PI3K/AKT1/mTOR, JAK2/STAT3 and HIF-1alpha/NF-kappaB signaling pathways. J Ethnopharmacol 314:116637. 10.1016/j.jep.2023.116637 37187363 10.1016/j.jep.2023.116637

[CR16] Nogales C, Mamdouh ZM, List M, Kiel C, Casas AI, Schmidt HHHW (2022) Network pharmacology: curing causal mechanisms instead of treating symptoms. Trends Pharmacol Sci 2:136–150. 10.1016/j.tips.2021.11.004 10.1016/j.tips.2021.11.004 34895945

[CR17] Osei E, Leila M, Hsieh A, Warner S, Al-Fouadi M, Wang M, Cole D, Maksym G, Hallstrand T, Timens W, Brandsma C, Heijink I, Hackett T (2020) Epithelial-interleukin-1 inhibits collagen formation by airway fibroblasts: implications for asthma. Sci Rep 10:8721. 10.1038/s41598-020-65567-z 32457454 10.1038/s41598-020-65567-z PMC7250866

[CR18] Peng S, Gao J, Liu W, Jiang C, Yang X, Sun Y, Guo W, Xu Q (2016) Andrographolide ameliorates OVA-induced lung injury in mice by suppressing ROS-mediated NF-κB signaling and NLRP3 inflammasome activation. Oncotarget 49:80262–80274. 10.18632/oncotarget.12918 10.18632/oncotarget.12918 PMC5348318 27793052

[CR19] Percie du Sert N, Hurst V, Ahluwalia A, Alam S, Avey MT, Baker M, Browne WJ, Clark A, Cuthill IC, Dirnagl U, Emerson M, Garner P, Holgate ST, Howells DW, Karp NA, Lazic SE, Lidster K, MacCallum CJ, Macleod M, Pearl EJ, Petersen OH, Rawle F, Reynolds P, Rooney K, Sena ES, Silberberg SD, Steckler T, Würbel H (2020) The ARRIVE guidelines 2.0: updated guidelines for reporting animal research. PLoS Biol. 10.1371/journal.pbio.3000410 32663219 10.1371/journal.pbio.3000410 PMC7360023

[CR20] Peters MC, McGrath KW, Hawkins GA, Hastie AT, Levy BD, Israel E, Phillips BR, Mauger DT, Comhair SA, Erzurum SC, Johansson MW, Jarjour NN, Coverstone AM, Castro M, Holguin F, Wenzel SE, Woodruff PG, Bleecker ER, Fahy JV (2016) Plasma interleukin-6 concentrations, metabolic dysfunction, and asthma severity: a cross-sectional analysis of two cohorts. Lancet Respir Med 7:574–584. 10.1016/S2213-2600(16)30048-0 10.1016/S2213-2600(16)30048-0 PMC5007068 27283230

[CR21] Raj JP, Maurya MR, Nair N, Marfatia H, Hadaye R, Gogtay NJ (2023) Efficacy and safety of AP-Bio® (KalmCold®) in participants with uncomplicated upper respiratory tract viral infection (common cold)—a phase III, double-blind, parallel group, randomized placebo-controlled trial. Complement Ther Med 73:102934. 10.1016/j.ctim.2023.102934 36842634 10.1016/j.ctim.2023.102934

[CR22] Ramakrishnan RK, Heialy SA, Hamid Q (2019) Role of IL-17 in asthma pathogenesis and its implications for the clinic. Expert Rev Respir Med 11:1057–1068. 10.1080/17476348.2019.1666002 10.1080/17476348.2019.1666002 31498708

[CR23] Ratiani L, Pachkoria E, Mamageishvili N, Shengelia R, Hovhannisyan A, Panossian A (2022) Efficacy of Kan Jang® in patients with mild COVID-19: interim analysis of a randomized, quadruple-blind, placebo-controlled trial. Pharmaceuticals 8:1013. 10.3390/ph15081013 10.3390/ph15081013 PMC9415141 36015163

[CR24] Reber LL, Daubeuf F, Nemska S, Frossard N (2012) The AGC kinase inhibitor H89 attenuates airway inflammation in mouse models of asthma. PLoS ONE 11:e49512. 10.1371/journal.pone.0049512 10.1371/journal.pone.0049512 PMC3506657 23189147

[CR25] Selvaraj K, Devi RG, Selvaraj J, Priya AJ (2022) In vitro anti-inflammatory and wound healing properties of *Andrographis echioides* and Andrographis paniculate. Bioinformation 18:331–336. 10.6026/97320630018331 36909694 10.6026/97320630018331 PMC9997496

[CR26] Sulaiman I, Tan K, Mohtarrudin N, Lim JCW, Stanslas J (2018a) Andrographolide prevented toluene diisocyanate-induced occupational asthma and aberrant airway E-cadherin distribution via p38 MAPK-dependent Nrf2 induction. Pulm Pharmacol Ther 53:39–51. 10.1016/j.pupt.2018.09.008 30244166 10.1016/j.pupt.2018.09.008

[CR27] Sulaiman I, Tan K, Mohtarrudin N, Lim JCW, Stanslas J (2018b) Andrographolide prevented toluene diisocyanate-induced occupational asthma and aberrant airway E-cadherin distribution via p38 MAPK-dependent Nrf2 induction. Pulm Pharmacol Ther 53:39–51. 10.1016/j.pupt.2018.09.008 30244166 10.1016/j.pupt.2018.09.008

[CR28] Sulaiman I, Okwuofu EO, Mohtarrudin N, Lim JCW, Stanslas J (2024) An *Andrographis paniculata* Burm Nees. extract standardized for three main Andrographolides prevents house dust mite-induced airway inflammation, remodeling, and hyperreactivity by regulating Th1/Th2 gene expression in mice. J Ethnopharmacol 319:117082. 10.1016/j.jep.2023.117082 37652197 10.1016/j.jep.2023.117082

[CR29] Verstraete K, Peelman F, Braun H, Lopez J, Rompaey DV, Dansercoer A, Vandenberghe I, Pauwels K, Tavernier J, Lambrecht BN, Hammad H, Winter HD, Beyaert R, Lippens G, Savvides SN (2017) Structure and antagonism of the receptor complex mediated by human TSLP in allergy and asthma. Nat Commun 8:14937. 10.1038/ncomms14937 28368013 10.1038/ncomms14937 PMC5382266

[CR30] Xu C, Huang H, Zou H, Zhao Y, Liu L, Chai R, Zhang J (2024) The miR-9–5p/KLF5/IL-1beta axis regulates airway smooth muscle cell proliferation and apoptosis to aggravate airway remodeling and inflammation in asthma. Biochem Genet 62:3996–4010. 10.1007/s10528-023-10640-1 38267617 10.1007/s10528-023-10640-1

[CR31] Yan X, Tong X, Jia Y, Zhao Y, Zhang Q, Hu M, Li X, Li B, Ming X, Xie Y, Wu X, Yu X, Qu L, Xiong L, Huang F, Nie J (2024) Baiheqingjin formula reduces inflammation in mice with asthma by inhibiting the PI3K/AKT/NF-kappab signaling pathway. J Ethnopharmacol 321:117565. 10.1016/j.jep.2023.117565 38081397 10.1016/j.jep.2023.117565

[CR32] Yang D, Li Y, Liu T, Yang L, He L, Huang T, Zhang L, Luo J, Liu C (2023) IL-1β promotes IL-17A production of ILC3s to aggravate neutrophilic airway inflammation in mice. Immunology. 10.1111/imm.13644 36988516 10.1111/imm.13644

[CR33] Yu Q, Shi Y, Shu C, Ding X, Zhu S, Shen Z, Lou Y (2021) Andrographolide inhibition of Th17-regulated cytokines and JAK1/STAT3 signaling in OVA-stimulated asthma in mice. Evid-Based Complement Alternat Med. 10.1155/2021/6862073 34194525 10.1155/2021/6862073 PMC8181172

[CR34] Zhu T, Wu X, Zhang W, Xiao M (2015) Glucagon like peptide-1 (GLP-1) modulates OVA-induced airway inflammation and mucus secretion involving a protein kinase A (PKA)-dependent nuclear factor-κB (NF-κB) signaling pathway in mice. Int J Mol Sci 9:20195–20211. 10.3390/ijms160920195 10.3390/ijms160920195 PMC4613197 26343632

